# Brain structural and functional damage network localization of COVID-19 survivors

**DOI:** 10.3389/fneur.2026.1766985

**Published:** 2026-04-01

**Authors:** Junyu Wu, Zhuqing Zhang, Xuejun Liu, Chunlei Zhang, Kai Huang, Han Zhao

**Affiliations:** Department of Radiology, The Affiliated Hospital of Qingdao University, Qingdao, China

**Keywords:** brain network, clinical treatment, COVID-19, functional connectivity, functional magnetic resonance imaging

## Abstract

**Purpose:**

Neuroimaging studies exploring structural and functional brain changes of COVID-19 survivors have yielded regionally inconsistent findings. Although there is an increasing agreement that diseases are more accurately mapped to distributed neural network than to discrete brain areas, research examining network-level localization of structural and functional deficits in COVID-19 survivors remains limited.

**Method:**

To bridge this gap, we first pinpointed sites of structural and functional impairment in COVID-19 survivors, drawing on 19 studies comprising 23 contrasts across a cohort of 703 survivors and 596 healthy controls. Using connectivity-based mapping, we projected these identified regions onto large-scale resting-state fMRI datasets to reconstruct a coordinated brain network associated with neurological abnormalities in COVID-19 survivors.

**Results:**

In COVID-19 survivors, structural and functional alternations were mapped to a widely distributed brain network, primarily involving the default mode and limbic systems.

**Conclusion:**

Our results reveal both common and distinct neural correlates underlying structural and functional impairments among COVID-19 survivors. These insights not only elucidate the neuropathology of the disease through a network-based framework but also support the development of therapeutic interventions for affected individuals.

## Introduction

Since the COVID-19 pandemic emerged in early 2020, millions of individuals have been infected with the SARS-CoV-2 virus (1, 2). A large number of these patients have experienced a wide range of symptoms, both during the acute phase of illness and as part of post-COVID condition. These symptoms include olfactory dysfunction, chronic fatigue, cognitive and attentional difficulties, sleep problems, depressive symptoms, post-traumatic stress disorder (PTSD), and even increased risks of self-harm and suicidal thoughts (3–7). More and more neuroimaging studies and clinical observations have revealed significant changes in brain structure and function after SARS-CoV-2 infection, emphasizing the urgent need to deepen research into its neurotropic effects and long-term neurological consequences (8).

Thanks to advances in neuroimaging techniques, especially multimodal magnetic resonance imaging (MRI), our ability to conduct psychiatric and neurological research have been greatly boosted (9). Utilizing these methodologies, many studies have shown structural and functional abnormalities in COVID-19 survivors (10–14, 68). To align results across different imaging modalities and task paradigms, researchers conducted an integrative meta-analysis. The meta-analysis found that, compared to healthy controls, individuals with a history of COVID-19 exhibited reduced neural activity in the right superior temporal gyrus, left insula, right orbitofrontal cortex and left cerebellum. Structurally, decreased gray matter volume (GMV) was found in the bilateral anterior cingulate cortex/medial prefrontal cortex and left cerebellum, while increased GMV was observed in the bilateral amygdala (15). These varied results support the growing agreement that diseases are better linked to distributed brain networks than to isolated brain regions (16, 17).

Traditional investigations of brain-behavior correlations have typically attributed neuropsychiatric symptoms to specific brain areas. However, there is a growing that the disorders are more accurately mapped to large-scale neural circuits rather than isolated anatomical structures (18, 19). In this study, we used an innovative computational technique—functional connectivity network mapping (FCNM). FCNM can integrates brain locations of interest with comprehensive human brain connectome data (9, 14, 20–23). FCNM represents a novel and efficient methodology that has been successfully applied to elucidate neurological and psychiatric symptoms, which is difficult to explain by traditional means (17, 24–32). Despite these advances, research examining the network-level localization of structural and functional impairments in COVID-19 survivors remains limited.

**Figure 1:**
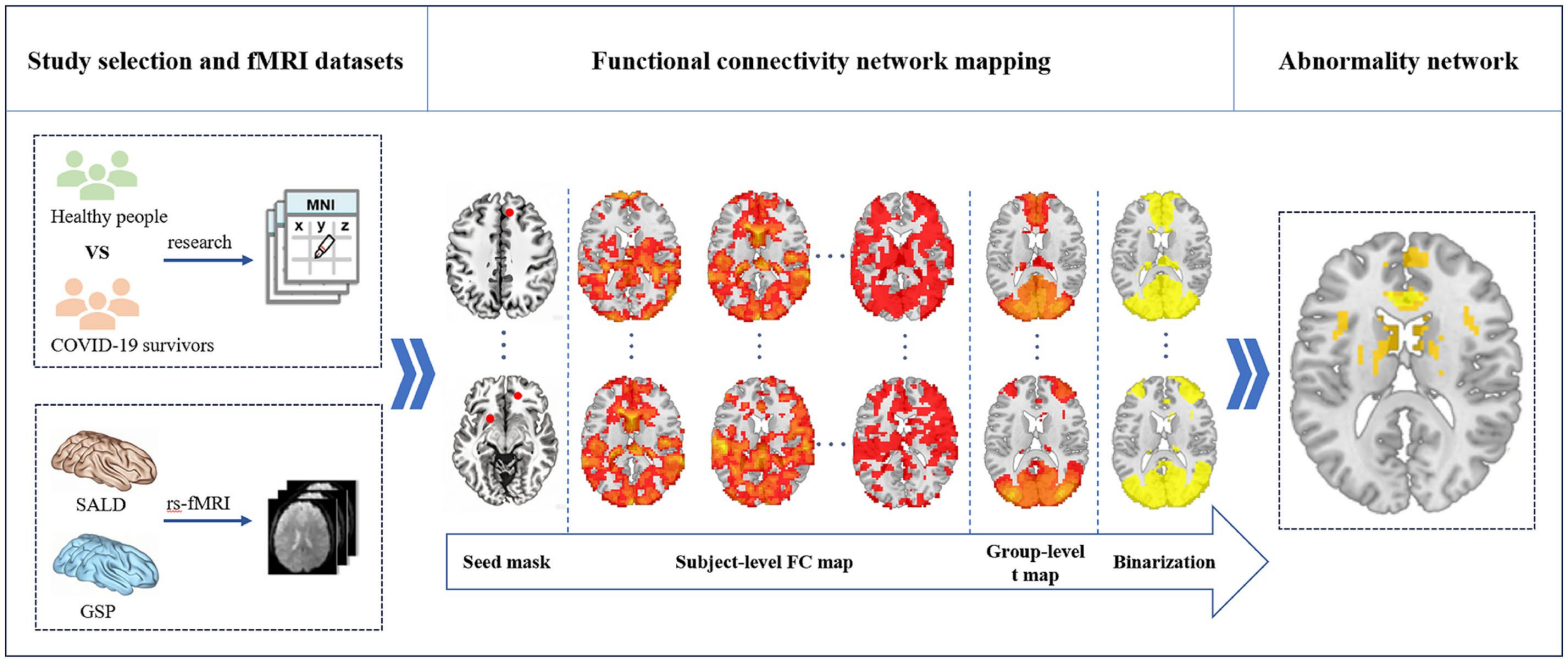
Study design and analysis pipeline. We initially synthesized published literature to identify brain alterations in structure and function between COVID-19 and healthy individuals. By integrating these affected brain locations with large-scale discovery (Southwest University Adult Lifespan Dataset [SALD]) and validation (GSP, the Brain Genomics Superstruct Project) resting state functional magnetic resonance imaging (rs-fMRI) datasets, we then adopted the functional connectivity (FC) network mapping approach to construct a COVID-19 survivors’ brain damage network. Specifically, a combined seed mask specific to each contrast was generated by creating spheres centered at its respective coordinates and merging them. Subsequently, utilizing the preprocessed resting-state fMRI data, we computed participant-level contrast seed-to-whole-brain FC maps. Third, these individual FC maps were then subjected to voxel-wise one-sample *t*-tests to identify brain regions exhibiting significant functional connectivity with each contrast seed at the group level. Fourth, these individual FC maps were then subjected to voxel-wise one-sample *t*-tests to identify brain regions exhibiting significant functional connectivity with each contrast seed at the group level. Finally, the binarized maps of COVID-19 survivors were overlaid to produce a network probability map, which were thresholded at 60% to yield brain structural and functional changes network location of COVID-19 survivors. SALD, Southwest University Adult Lifespan Dataset; FC, functional connectivity; rs-fMRI, resting-state functional magnetic resonance imaging; GSP, the Brain Genomics Superstruct Project.

To reconcile the heterogeneity in prior neuroimaging findings regarding COVID-19 survivors’ brains, this study adopted a network perspective using the FCNM approach. We first integrated reported evidence of cerebral structural and functional changes in COVID-19 survivors. We then applied FCNM method to link these identified regions with large-scale discovery and validation resting-state magnetic resonance imaging (rs-fMRI) datasets, thereby reconstructing a coordinated brain network associated with COVID-19 survivors. A schematic overview of the study workflow and analytical procedure is presented in Figure 1.

## Materials and methods

### Study search and selection

We conducted a systematic search of PubMed and Web of Science for studies published before December 2023 that compared brain structure and function between COVID-19 survivors and healthy controls. More details about the literature search strategy are available in the Supplementary material. Because individual studies often present multiple independent comparisons, we centered our analysis on specific contrast results rather than treating each study as a single unit. We manually screened eligible studies according to predefined criteria, and the study selection procedure is summarized in Supplementary Figure S1. The peak voxel coordinates from significant clusters were extracted for every contrast. A spatial transformation was applied to standardize all coordinates to the Montreal Neurological Institute (MNI) template, which included those originally reported in the Talairach atlas. The methodology for this systematic review was preregistered in PROSPERO (https://www.crd.york.ac.uk/PROSPERO/, registration number: CRD420250654127).

### Discovery and validation datasets

We utilized the Southwest University Adult Lifespan Dataset (SALD) as the discovery cohort (33) and the Brain Genomics Superstruct Project (GSP) as an independent validation dataset (34). The SALD dataset includes 328 healthy adults (206 female; mean age 37.74 [13.76] years). Exclusion criteria for SALD participants comprised contraindications to MRI, current psychiatric or neurological disorders, use of psychoactive substances within the preceding 3 months, pregnancy, and a history of significant traumatic brain injury. Detailed sample characteristics have been reported in another publication (22). The GSP dataset consists of 1,430 healthy adults (837 female; mean age 21.58 [2.93] years), with left-handed and ambidextrous individuals excluded. Notably, we restricted analyses to participants between 18 and 60 years of age to reduce potential confounding effects from ongoing brain development and aging-related degeneration. Detailed demographic information for the discovery and validation datasets is presented in Supplementary Table S1. This study was approved by the institutional Ethics Committee of the Affiliated Hospital of Qingdao University (QYFY WZLL 30682) and was conducted in accordance with the Declaration of Helsinki.

### Acquisition and preprocessing of fMRI data

Rs-fMRI data for both the SALD and GSP datasets were acquired on 3 T Siemens Trio scanners. The specific imaging parameters for each dataset are provided in Supplementary Table S2. Participants should be excluded if their scans exhibited obvious artifacts or incomplete brain coverage.

Preprocessing of rs-fMRI data was conducted using SPM12 (https://www.fil.ion.ucl.ac.uk/spm/) and DPABI (https://rfmri.org/DPABI) according to a validated pipeline (9, 20–23, 35, 36). The initial 10 volumes for per subject were disregarded to allow for magnetic field stabilization and participant acclimation. Theses subsequent volumes were processed with slice-timing correction to adjust for the time differences in slice acquisition, followed by rigid-body realignment for head motion correction. Head motion was quantified by calculating the translational and rotational displacement for per volume. All included participants exhibited maximum translational and rotational displacements of less than 2 mm and 2°, respectively. Framewise displacement was also calculated to assess volume-to-volume changes in head position. The following nuisance covariates were removed via regression, including linear drift, motion parameters derived from the Friston-24 model, high-motion time points with framewise displacement above 0.5 mm, and global, white matter, and cerebrospinal fluid signals. Global signal regression was implemented to improve sensitivity to system-selective connectivity patterns and increase correspondence with anatomical pathways (37). The data were then temporally band-pass filtered (0.01–0.1 Hz) to isolate low-frequency fluctuations. During spatial normalization phase, structural images from each participant were first aligned to their mean functional images, followed by segmentation and transformation into MNI space using a diffeomorphic approach based on exponentiated Lie algebra (38). The spatially filtered functional volumes were subsequently normalized to the identical stereotactic space by applying the obtained deformation parameters and resampled to 3-mm isotropic voxels. Lastly, all data underwent spatial smoothing with a 6 × 6 × 6 mm^3^ Gaussian kernel at full width at half maximum.

### Functional connectivity network mapping

To identify the network associated with structural and functional impairment in COVID-19 survivors, we employed the FCNM method based on previously extracted neuroimaging coordinates (Figure 1). Firstly, 4-mm spherical regions of interest were created around each significant coordinate from a given contrast and merged into a composite seed mask. Secondly, using preprocessed rs-fMRI data obtained from the SALD dataset, we constructed whole-brain functional connectivity (FC) maps for each participant. Specifically, Pearson’s correlation coefficients were computed between time courses of the composite seed and every voxel across the brain. These correlation values were then converted to z-scores using Fisher’s transformation to improve normality. Thirdly, a voxel-wise one-sample t-test was performed across all 328 individual FC maps to identify brain regions exhibiting significant positive FC with the composite seed region. Analyses were restricted to positive correlations due to ongoing discussion regarding the biological interpretation of negative FC (37, 39). Fourthly, group-level *t*-statistic maps were thresholded and binarized. A voxel-wise false discovery rate (FDR) correction was applied to control for multiple comparisons, with a significance threshold set at *p* < 0.05. Finally, binarized maps from all relevant contrasts were overlaid to generate a consensus probability map, which was thresholded at 60% overlap to delineate the dysfunctional network associated with COVID-19 survivors.

### Relation to canonical brain networks

To enhance interpretability of our findings, we evaluated the spatial overlap between the abnormal network observed in COVID-19 survivors and seven well-established canonical functional networks. These cortical networks involve ventral attention, dorsal attention, somatomotor, visual, frontoparietal, limbic, and default mode networks (DMN) in light of the cited study (40). We calculated spatial overlap as the share of voxels in the COVID-19-related network that overlapped with each canonical functional network.

### Validation analyses

To confirm the reliability of our results, we implemented multiple verification measures. For one, we assessed the generalizability of our findings by replicating the key FCNM procedure in the GSP dataset. For another, we tested the robustness of our results to seed definition settings by repeating the analysis with 1 mm and 7 mm radius spherical seeds. Additionally, to validate the specificity of the identified COVID-19-related brain network and rule out random artifacts, we performed a control analysis using randomly generated brain coordinates. We first created a set of random intracranial coordinates that matched the number and spatial distribution of the original seed coordinates used in the primary FCNM analysis. Using these random coordinates, we re-ran the entire FCNM pipeline with identical parameters as the main analysis.

## Results

### Included studies

Based on an extensive review of literature, we selected 19 studies encompassing 23 distinct experimental contrasts in our analyses. These studies included a total of 703 COVID-19 survivors and 596 healthy controls matched for demographic characteristics. Extended information regarding sample composition and image acquisition settings can be found in Supplementary Table S3.

### Survivors’ dysfunctional network

**Figure 2:**
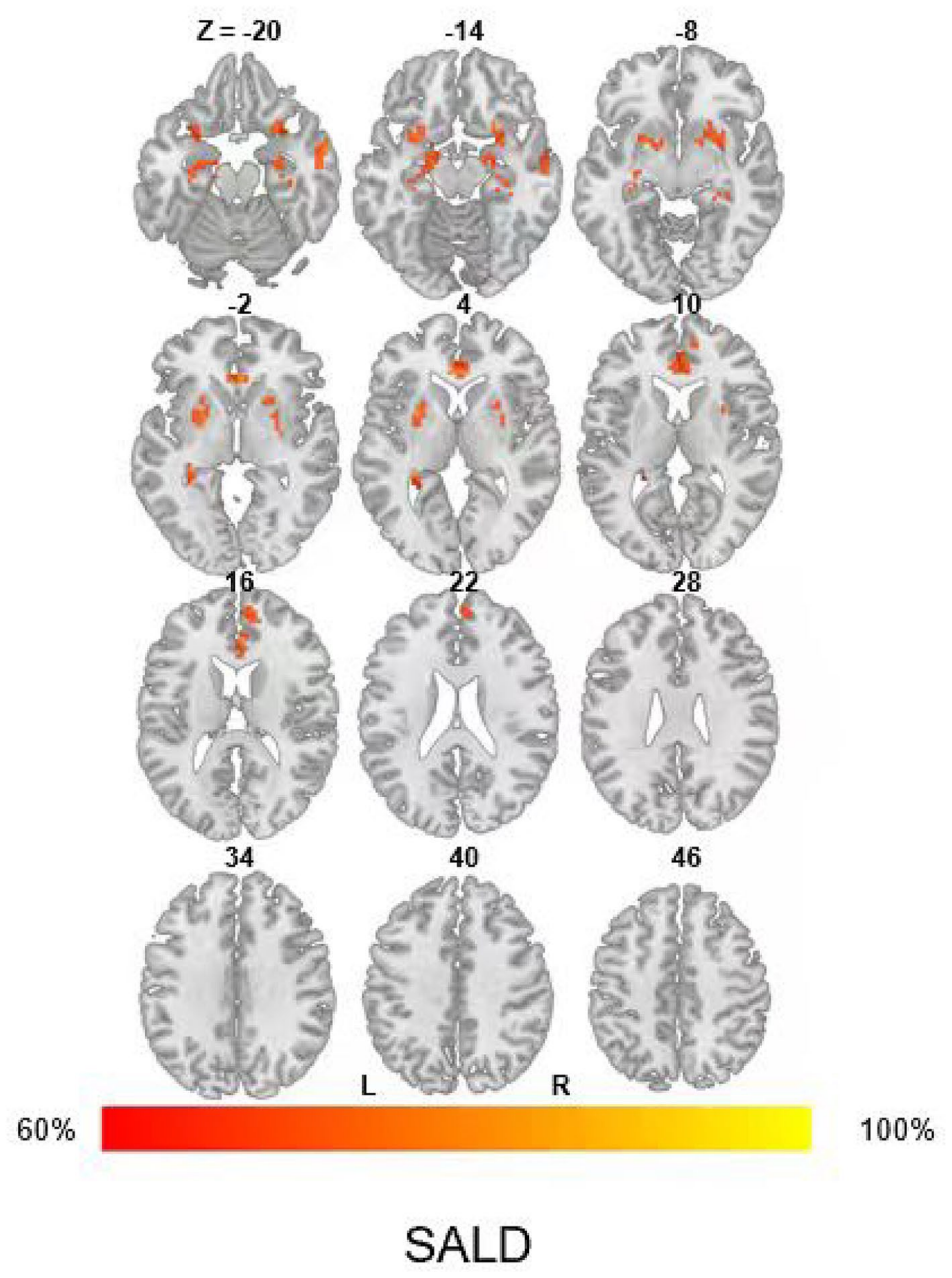
COVID-19 survivors’ dysfunctional networks. The dysfunctional network is presented as network probability maps thresholded at 60%, showing brain regions functionally connected to more than 60% of the contrast seeds. SALD, Southwest University Adult Lifespan Dataset.

**Figure 3:**
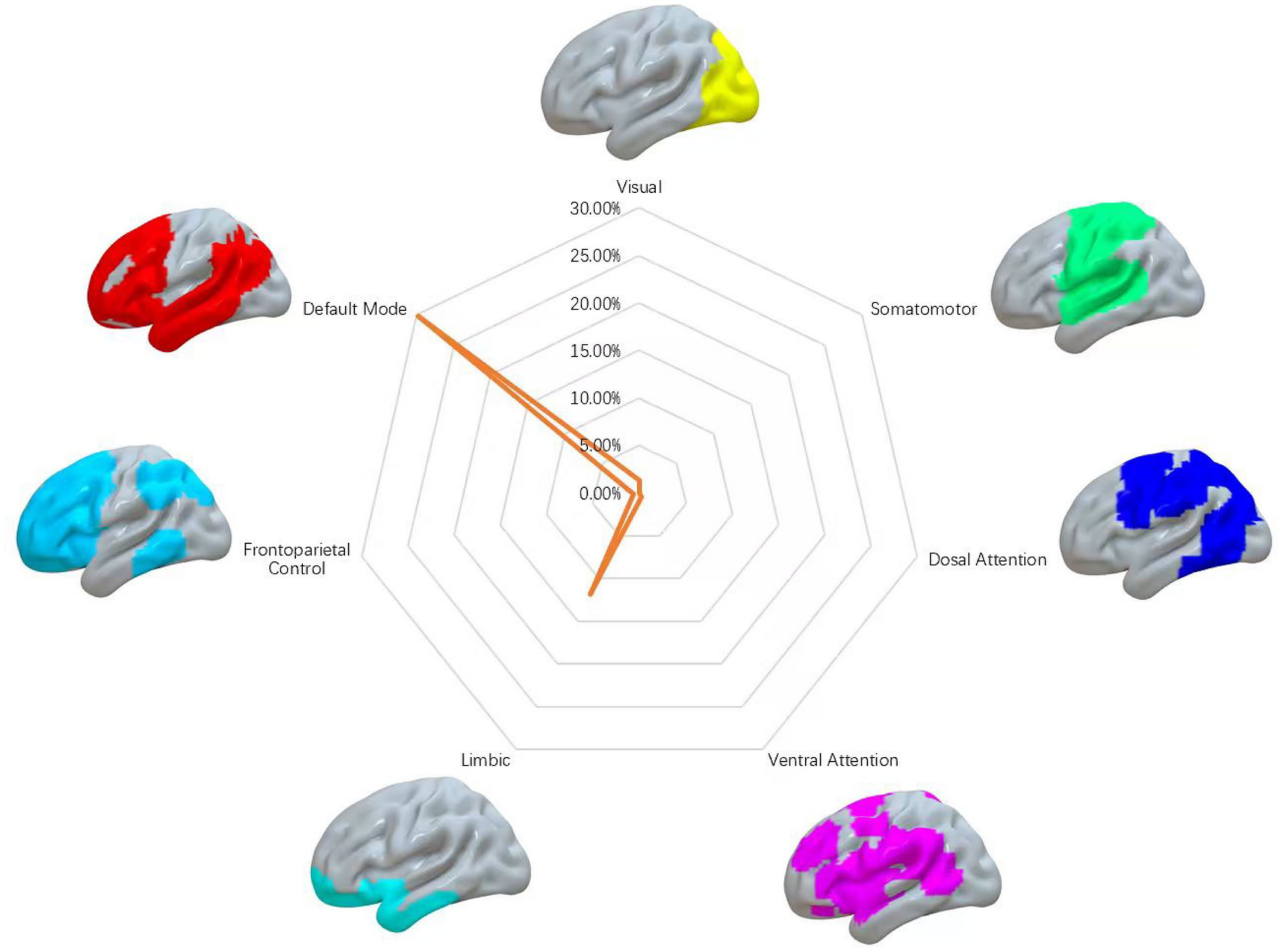
COVID-19 survivors’ dysfunctional networks in relation to canonical brain networks. Polar plots illustrate the proportion of overlapping voxels between survivors’ dysfunctional network and a canonical network to all voxels within the corresponding canonical network.

Brain network alterations observed in COVID-19 survivors spread across a range of different regions. Affected areas encompassed the bilateral temporal cortex (superior and middle temporal gyrus), medial prefrontal cortex, olfactory gyrus, hippocampus, uncus, and subcortical structures (putamen, pallidum, and thalamus) (Figure 2). When mapped onto canonical networks, the survivors’ dysfunctional network was strongly linked to the DMN (overlapping proportion: 29.78%), and limbic network (11.91%) (Figure 3).

### Validation analyses

The survivors’ abnormal network patterns obtained from the discovery SALD dataset closely matched those identified in the validation GSP dataset (Supplementary Figure S2). Furthermore, we repeated FCNM analysis using different seed sizes, including 1-mm and 7-mm radius spheres. These repeated analyses yielded network maps that were highly consistent with those generated using the original 4-mm seeds (Supplementary Figure S3, S4). Additionally, we conducted a control FCNM analysis using randomly generated brain coordinates that matched the number and spatial distribution of the original seed coordinates, and this control analysis did not produce any significant connectivity networks. Above results demonstrated that our core findings were stable despite variations in dataset and methodological settings, and confirmed that the identified COVID-19-related brain network was not a random artifact.

## Discussion

Using the novel FCNM method with the large-scale human connectome dataset, our study identified network-specific neural alterations in patients recovering from COVID-19, building on previously reported patterns of cerebral structural and functional impairment. We found that the affected brain areas form a widely distributed system, primarily involving the DMN and limbic networks. These discoveries may reconcile inconsistent neuroimaging results, deepen our understanding of the neurobiological mechanisms of COVID-19 survivors from a network view, and ultimately provide more precise treatment approaches.

Neuroimaging is a widely used tool to study brain structure and function in psychiatric disorders and has produced many promising findings (41). However, the poor reproducibility of most results is a major barrier to applying these advances into clinical practice (42). The irreproducibility issue of psychiatric neuroimaging may stem from multiple factors, such as small sample sizes that lack statistical power, clinically heterogeneous participant groups, highly flexible experimental designs, inconsistent analytical workflows across research teams, and the use of poorly controlled statistical approaches (43). To address the above problems, researchers often use coordinate-based meta-analyses to combine findings from multiple studies on psychiatric conditions to identify consistent regional brain abnormalities. However, it is increasingly acknowledged that disease-related processes do not affect just one area but rather spread through connected brain networks, which means that mental illnesses are better understood from a network point of view (18). Consequently, instead of focusing only on specific brain regions, we now link diseases to neuroanatomy toward a more integrated network-based perspective. This methodological change has been made possible mainly by the advancement of the FCNM technique. This approach enables researchers to map symptoms, diseases, or cognitive functions to brain networks by combining areas showing lesions, structural damage, functional abnormalities, or activation with large-scale maps of brain connectivity (28). The FCNM method operates on the premise that the neural pathology underlying a given disorder may present in varied anatomical sites, yet engages in the same neural circuit. This approach has revealed common brain networks associated with illnesses such as depression, schizophrenia, and Alzheimer’s disease (9, 20–23, 25, 26, 30–32, 44–46). Following this lead, we used the FCNM approach to examine how these abnormalities are organized at the level of brain networks.

We found that the structural and functional damage network in COVID-19 survivors consisted of widely distributed brain regions primarily including the bilateral temporal cortex (superior and middle temporal gyrus), the medial prefrontal cortex, olfactory gyrus, hippocampus, uncus, and subcortical structures (putamen, pallidum, and thalamus). The superior anterior temporal cortex plays a central role in social cognition, integrating abstract conceptual knowledge necessary for understanding social behavior. Studies further suggest that people with social anxiety disorder tend to have reduced volume in the superior temporal gyrus relative to healthy control subjects (47). Studies of brain injuries show that damage to specific parts of the temporal lobes can lead to clear problems with several high-level cognitive abilities, including recognizing objects, understanding speech, naming items, reading, and spelling (48). All of these can lead to “brain fog.” The anterior cingulate cortex plays a role in evaluating motivational relevance and identifying negative consequences (49, 50). It acts as an integration hub for sensory, cognitive, and emotional signals, and this function is crucial for self-regulatory processes (51). Therefore, damage to the anterior cingulate cortex may be related to the occurrence of anxiety, depression, and even suicidal behavior in COVID-19 survivors (52). The olfactory pathway originates in the olfactory bulb and is related to the anterior olfactory nucleus, piriform cortex, and entorhinal cortices. Several secondary regions are involved in higher-order olfactory processing, such as the insula, hypothalamus, and hippocampus (53). The piriform cortex is sometimes referred to as the lateral olfactory gyrus, and in this sense, it can be considered to belong to the concept of the “olfactory gyrus” in a broad sense. The uncus is part of the olfactory cortex and receives information about odors. More and more evidence suggests that abnormalities in the olfactory gyrus, hippocampus, and uncus are linked to hyposmia/anosmia in COVID-19 survivors (54, 55). Additionally, there is evidence showing that perfumers who imagine smells and sommeliers who taste wine display special activation patterns in olfactory processing areas (piriform cortex, orbitofrontal cortex), as well as the hippocampus (56). The thalamus and basal ganglia, which includes putamen and pallidum, form circuits with cortex supporting cognitive, motor, and emotional domains (57). Moreover, the basal ganglia and thalamus are rich in dopaminergic innervation (58), which is of great significance for motor control, emotional regulation and memory. If these regions are damaged, it may lead to PTSD, “brain fog,” anxiety, and depression (59).

Concerning canonical brain networks, the spatial distribution of structural and functional damage in COVID-19 survivors primarily implicated default mode and limbic networks. The DMN is critically involved in self-awareness, interpersonal understanding, recollection of personal experiences, and imagining future scenarios (22, 60). Growing research suggests that changes in the functional and structural integrity of the DMN are linked to impaired sense of smell, depressive symptoms, and deficits in cognitive performance (61–63). The limbic system comprises interconnected brain regions that help link emotions, bodily feelings, and motivational drives to our thoughts and actions (64). Important subcortical parts of this system include the amygdala, mammillary bodies, hypothalamus, some thalamic nuclei and the ventral striatum. Research has shown that when this network is damaged or not working properly, it is associated with neuropsychiatric disorders such as PTSD, anxiety disorders, and autism (17, 45, 65, 70).

Our study has several limitations. First, due to the limited number of studies on COVID-19, our research did not distinguish between structural and functional abnormalities. Although structural brain abnormalities and functional network disruptions are closely interrelated in neuropathological processes, the combined application of structural and functional data in FCNM analysis lacks direct methodological validation. This constitutes a major methodological limitation of our study, and it will be necessary to conduct further dedicated methodological investigations in the future to validate the feasibility of this integrative strategy. Second, the rs-fMRI scans were acquired from healthy adults to study which brain networks are affected in COVID-19 survivors. It seems to be better to use a control group that closely matches survivors in age, health, and background, which helps improve the reliability of findings by reducing the impact of differences that are not related to illness itself. Nevertheless, empirical evidence shows that the choice of sample has minimal influence on the outcomes of network localization (24, 34, 66, 69). Third, the design of our investigation was retrospective, relying on synthesized coordinate data from previously published research to reduce selection biases. Future studies adopting a prospective design are necessary to provide empirical verification of our results. Fourth, a liberal overlap threshold was adopted at 60% to delineate brain regions demonstrating neural connections with 60% of the seeds generated for contrast. The studies included in our analyses varied in terms of statistical power and methodological approaches, which could potentially limit our ability to detect a consistent brain network. Future research that addresses these sources of variability may enhance the current methodological framework. Fifth, our analysis did not adjust for the differences in sample size and effect of seed size from each study. This is because the research community has not yet established a reliable method to handle these variables. We expect that future work will develop more advanced methods to investigate and resolve this limitation. Sixth, while FCNM has aided in identifying phenotype-related brain network abnormalities, conventional FCNM may converge on elementary topological properties of normative matrices rather than disease-specific alterations, due to reliance on repetitive resampling of fixed templates (67). To address these limitations, future FCNM applications may consider adopting data-driven inputs, integrating multi-dimensional validation, and refining algorithmic parameters, thereby enhancing the rigor of research findings. Seventh, the included studies demonstrated substantial heterogeneity across sample demographics, clinical characteristics, neuroimaging acquisition parameters, and analytical pipelines, which may potentially impact the findings of the present study (43). Notably, consistency across such heterogeneous studies does not necessarily confirm result robustness, as it may alternatively arise from shared methodological artifacts (67). Future large-scale investigations employing strictly homogeneous populations, standardized imaging protocols, and unified analytical approaches are therefore warranted to overcome this limitation and further validate the specificity and stability of the identified brain network alterations. Finally, partial regions in our identified structural and functional network overlapped with transdiagnostic brain hubs that are commonly detected across various neuropsychiatric disorders (67). Consequently, the clinical specificity of our findings might have been overstated, and the unique pathological features of COVID-19-related brain abnormalities should be interpreted with caution.

In summary, our study applied an innovative FCNM method and integrated large-scale connectome data to map distributed structural and functional brain changes in COVID-19 survivors onto a specific brain network. These results help harmonize previously inconsistent neuroimaging findings and advance our understanding of the neuropathology of COVID-19 through a network-based perspective. Moreover, the identification of brain damage networks in COVID-19 survivors could help develop targeted prevention and treatment approaches. Specifically, the brain network markers identified in these survivors may have translational value, which can help confirm targets for noninvasive neuromodulation techniques like transcranial magnetic stimulation and transcranial direct current stimulation. Meanwhile, these markers can help monitor progression of illness and evaluate efficacy of therapeutic strategies in COVID-19 survivors.

## Data Availability

The original contributions presented in the study are included in the article/Supplementary material, further inquiries can be directed to the corresponding author.

## Author’s note

The study was presented as network probability maps thresholded at 60%, showing brain regions functionally connected to more than 60% of the contrast seeds. SALD, Southwest University Adult Lifespan Dataset.
