# Supplementary material for: Brain structural and functional damage network localization of COVID-19 survivors
Source: Front Neurol. 2026 Apr 1;17:1766985. doi: 10.3389/fneur.2026.1766985 (PMC13079111; doi:10.3389/fneur.2026.1766985)
Supplement: Supplementary file 1 [file Data_sheet_1.docx]

**Full Title:**

**Brain structural and functional damage network localization of COVID-19 survivors**

**Junyu Wu^1#^, Zhuqing Zhang^1#^, Xuejun Liu^1^, Chunlei Zhang^1^, Kai Huang^1^, Han Zhao^1*^**

**Running Title:**

**Brain abnormality networks of COVID-19**

^1^Department of Radiology, The Affiliated Hospital of Qingdao University, Qingdao, China

**^#^**These authors contributed equally to this work.

***Correspondence to:**

Han Zhao, MD E-mail: [zhaohan42@qdu.edu.cn](mailto:zhaohan42@qdu.edu.cn)

Department of Radiology, The Affiliated Hospital of Qingdao University, Qingdao, 266000, China.

**Supplementary materials**

**Table S1.** Demographic information of the discovery and validation datasets

| **Dataset** | **Sample size** | **Age (years)** | **Gender (F/M)** | **FD (mm)** |
| --- | --- | --- | --- | --- |
| SALD | 328 | 37.74± 13.76 | 206/122 | 0.18 ± 0.12 |
| GSP | 1430 | 21.58 ± 2.93 | 837/593 | 0.12± 0.10 |

Age and FD are expressed as mean ± standard deviation. Abbreviations: SALD, Southwest University Adult Lifespan Dataset; F, female; FD, frame-wise displacement; M, male; GSP，Brain Genomics Superstruct Project.

**Table S2.** Resting-state fMRI parameters of the discovery and validation datasets

| **Parameters** | **SALD** | **GSP** |
| --- | --- | --- |
| Scanner | 3.0T Siemens Trio | 3.0T Siemens Trio |
| Sequence | GRE-EPI | GRE- EPI |
| TR (ms) | 2000 | 3000 |
| TE (ms) | 30 | 30 |
| FA (°) | 90 | 85 |
| FOV (mm^2^) | 220 × 220 | 216 × 216 |
| Matrix size | 64 × 64 | 72 × 72 |
| Slice thickness (mm) | 3 | 3 |
| Slice gap (mm) | 1 | 0 |
| Slices | 32 | 17 |
| Time points | 242 | 124 |

Abbreviations: AMUD, Anhui Medical University Dataset; EPI, echo planar imaging; FA, flip angle; fMRI, functional magnetic resonance imaging; FOV, field of view; GRE, gradient echo; GSP, the Brain Genomics Superstruct Project; SS, single shot; TE, echo time; TR, repetition time.

**Table S3.** Sample and imaging characteristics of the studies included in the COVID-19 analysis

| **Study** | **Demographic characteristic** | | | | | | | | | | | | | | | | | | | | | | | | | | **Imaging characteristic** | | | | | | |
| --- | --- | --- | --- | --- | --- | --- | --- | --- | --- | --- | --- | --- | --- | --- | --- | --- | --- | --- | --- | --- | --- | --- | --- | --- | --- | --- | --- | --- | --- | --- | --- | --- | --- |
|  | **Number/female** | | | | **Age(years)** | | | | | | | | | | | | **Education(years)** | | | | | | | | | | **screener** | | | | | | |
|  | **Survivors** | **Controls** | | | **Survivors** | | | | | | | **Controls** | | | | | | **Survivors** | | | | | **Controls** | | | |  |  |  |  |  |  |  |
| (Li, Liu et al. 2023) | 35/18 | | 36/17 | | NA | | | | | NA | | | | | | | | | | | NA NA | | | | | MRI | | | | | | |  |
| (Díez-Cirarda, Yus et al. 2023) | 86/57 | | 36/22 | | 50.71±11.20 | | | | | | | | | 49.33±15.99 | | | | | | | 14.20±2.34 15.43±3.28 | | | | | 3.0T MRI | | | | | | |  |
| (Goehringer, Bruyere et al. 2023) | 28/21 | | 28/21 | | 46.1±9.8 | | | | | | | 46.1±9.8 | | | | | | | | | NA NA | | | | |  | | | PET | |  |  |  |
| (Ajcevic, Iscra et al. 2023) | 24/15 | | 22/13 | | 53.0±14.5 | | | | | | | | | 54.8±9.1 | | | | | | | 14.3±3.2 14.9±3.0 | | | | |  | | | 3.0T MRI | | | |  |
| (Wingrove, Makaronidis et al. 2023) | 11/8 | | 18/9 | | 37.02±9.08 | | | | 38.89±11.39 | | | | | | | | | | |  | NA NA | | | | |  | | | 3.0T MRI | | | |  |
| (Kamasak, Ulcay et al. 2023) | 50/25 | | 50/25 | | 38.10±5.85 | | | | | | | | 38.75±6.16 | | | | | | |  | NA NA | | | | |  | | | 1.5T MRI | | | |  |
| (Du, Zhao et al. 2023) | 22/11  18/9 | | 27/20 | | 54.14±9.76  53.50±10.03 | | | | | | | | 50.81±11.48 | | | | | | |  | 13,45±3,57  13,56±3.87 | | | 12,56±3.99 3.0T MRI | | | | | | | | |  |
| (Chang, Ryan et al. 2023) | 29/19 | | 21/12 | | 42.4±12.3 | | | | | | | | | | | 41.5±12.2 | | | | | NA NA | | | | | 3.0T MRI | | | | | | |  |
| (Bispo, Brandao et al. 2022) | 56/36 | | 37/22 | | 37.2±9.4 40.2±11.8 | | | | | | | | | | | | | | | | 15.3±3.3 15.0±3.3 | | | | | 3.0T MRI | | | | | | |  |
| (Kim, Ji et al. 2022) | 11/5 | | 39/27 | | 41.5±13.4 41.9±12.6 | | | | | | | | | | | | | | | | | 17.0±2.8 16.0±2.0 | | | | | | | | 3.0T MRI | | |  |
| (Hafiz, Gandhi et al. 2022) | 46/15 | | 30/7 | | 34.63±11.54 | | | | | | 33.50±9.74 NA NA | | | | | | | | | | | | | | | 3.0T MRI | | | | | | |  |
| (Morand, Campion et al. 2022) | 7/6 | | 21/NA NA | | | | | NA NA NA PET | | | | | | | | | | | | | | | | | | | | | | | |  |  |
| (Tu, Zhang et al. 2021) | 47/33 | | 43/32 | | 51.8±11.3 | | | | | | | | | | | 52.0±11.0 | | | | | NA NA | | | | | 3.0T MRI | | | | | | |  |
| (Qin, Wu et al. 2021) | 19/12  32/16 | | 31/13 | | | 59.37±5.87  63.19±5.37 | | | | | | | | | 60.58±6.42 | | | | 11.05±3.12  10.84±2.67 | | | | | | 10.48±3.51 | | | | | 3.0T MRI | | |  |
| (Kas, Soret et al. 2021) | 7/3 | | | 32/NA | | | NA | | | | | | | | | NA | | | | | | NA NA | | | | | | PET | | | |  |  |
| (Du, Zhao et al. 2022) | 22/11 | | | 29/18 | | | 54.210±8。696 | | | | | | | | | 50.480±11.576 | | | | | | 13.420±3.610 12.720±4.088 | | | | | | | 3.0T MRI | | |  |  |
| (Donegani, Miceli et al. 2021) | 14/7 | | | 14/NA | | | NA | | | | | | | | | NA | | | | | | NA NA | | | | | | PET | | | |  |  |
| (Besteher, Machnik et al. 2022) | 30/17 | | | 20/10 | | | 47.5±11.5 | | | | | | | | | 42.95±13.41 | | | | | | 10.96±1.1 11±1.1 | | | | | | 3.0T MRI | | | |  |  |
| (Guedj, Campion et al. 2021) | 35/20 | | | 44/25 | | | 55.06±11.22 | | | | | | | | | 55.11±14.07 | | | | | | NA NA | | | | | | PET | | | |  |  |

Abbreviations: MRI, magnetic resonance imaging; NA, not applicable; PET, positron emission tomography; PSYRATS, psychotic symptom rating scales; SAPS, scale for the assessment of positive symptoms; SAPS-AH, scale for the assessment of positive symptoms-auditory hallucinations; SANS, scale for the assessment of negative

The following combination of search terms were used: (“corona virus disease-19” OR “COVID-19”) AND (“MRI” OR “fMRI” OR “magnetic resonance imaging” OR “PET” OR “positron emission tomography” OR “SPECT” OR “single photon emission computed tomography” OR “ASL” OR “arterial spin labeling” OR “neuroimaging” OR “FC” OR “functional connectivity” OR “ReHo” OR “regional homogeneity” OR “ALFF” OR “fALFF” OR “amplitude of low-frequency fluctuation*” OR “CBF” OR “blood flow” OR “glucose metabolism” OR “VBM” OR “voxel based morphometry” OR “DBM” OR “deformation based morphometry” OR “GMV” OR “gray matter” OR “grey matter”).


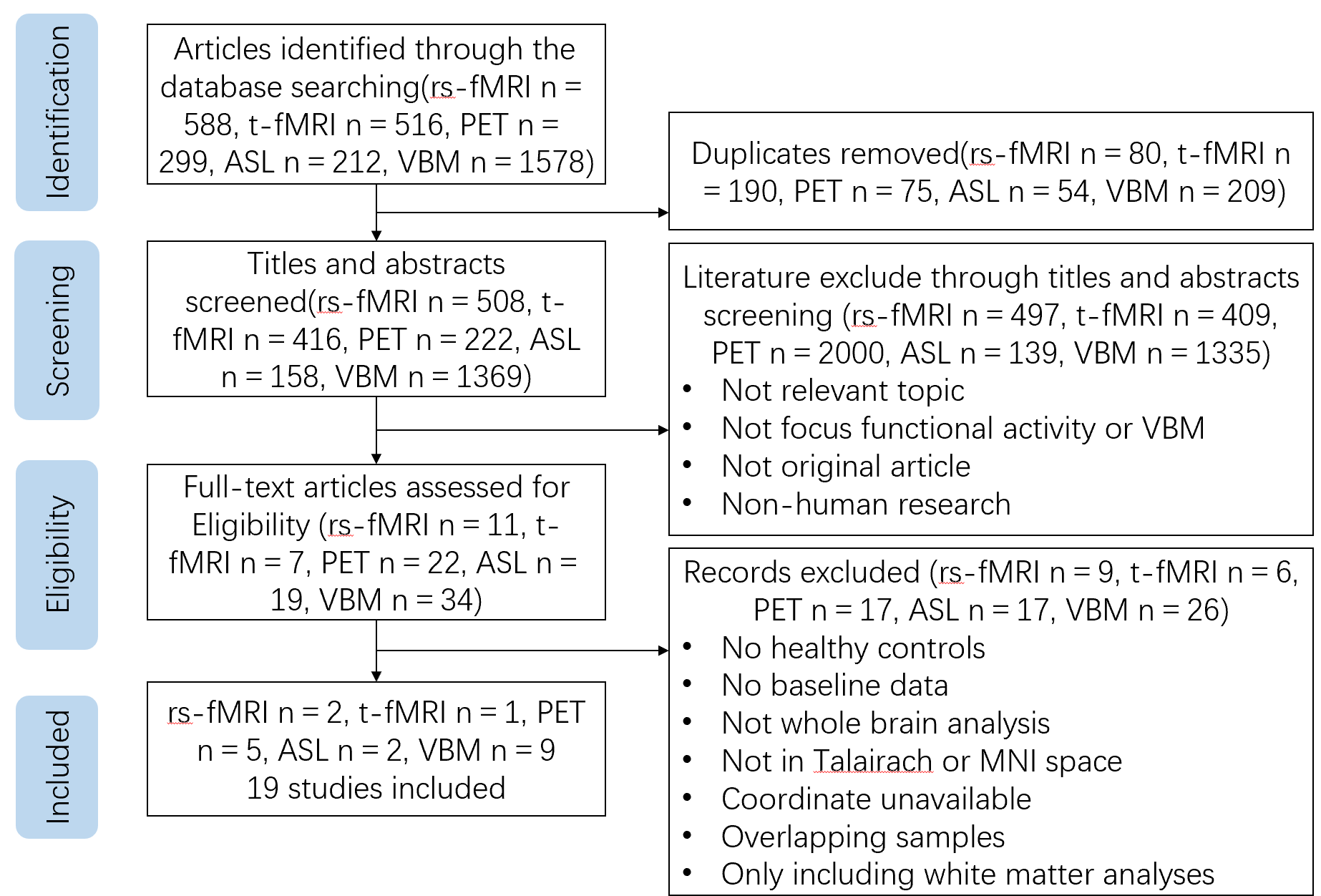


**Figure S1.** A flow diagram of the study selection process

Abbreviations: rs-fMRI, resting-state functional magnetic resonance imaging; t-fMRI, task-based functional magnetic resonance imaging; PET, positron emission tomography; ASL, arterial spin labelling; VBM, voxel-based morphometry.


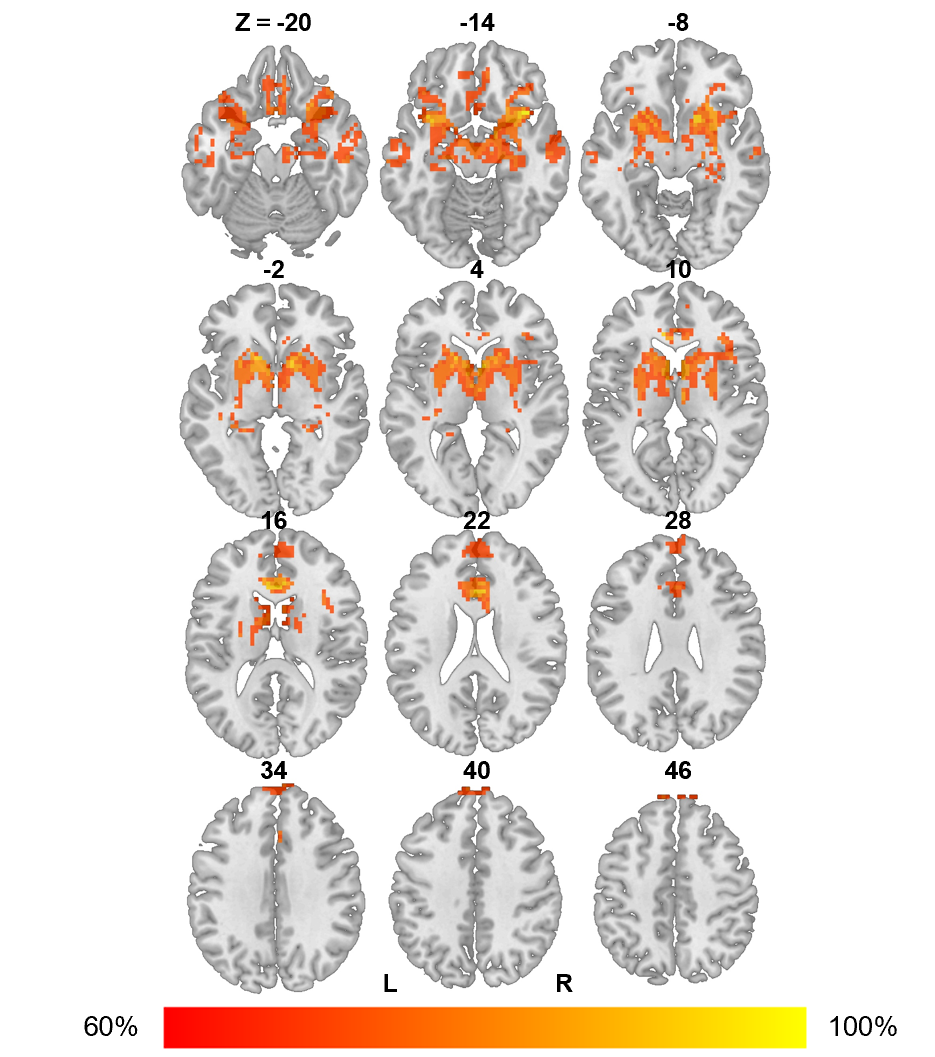


**Figure S2.** Brain structural and functional damage location of COVID-19 survivors derived from the GSP dataset. The dysfunctional network is presented as network probability maps thresholded at 60%, showing brain regions functionally connected to more than 60% of the contrast seeds. Abbreviations: L, left; R, right; GSP, the Brain Genomics Superstruct Project.


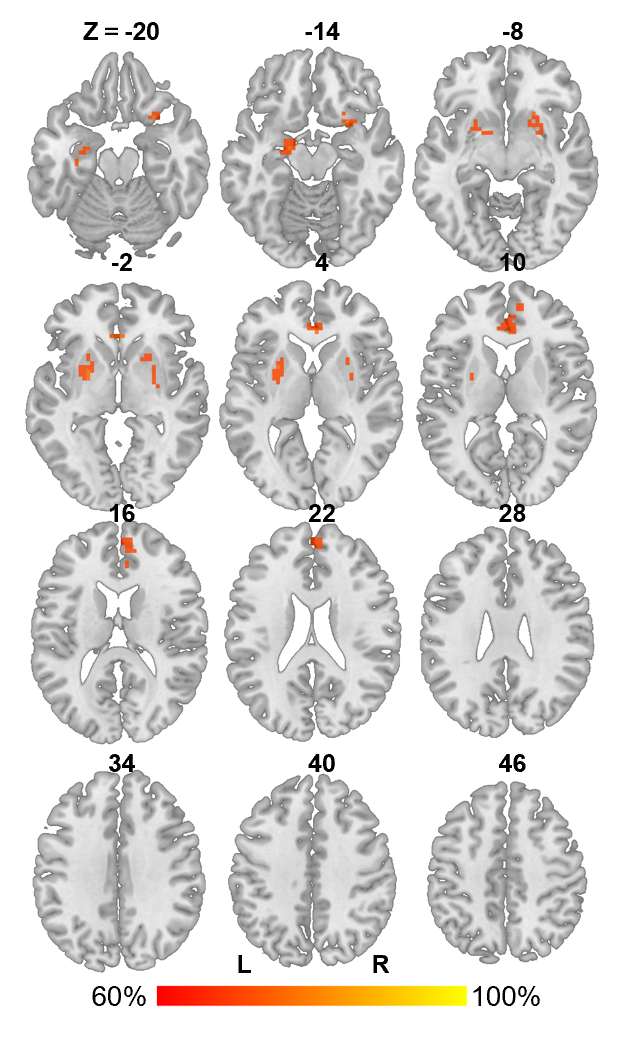


**Figure S3.** The COVID-19 survivors’ dysfunctional networks based on 1-mm radius sphere. The dysfunctional network is presented as network probability maps thresholded at 60%, showing brain regions functionally connected to more than 60% of the contrast seeds. Abbreviations: L, left; R, right; SALD, the Southwest University Adult Lifespan Dataset.


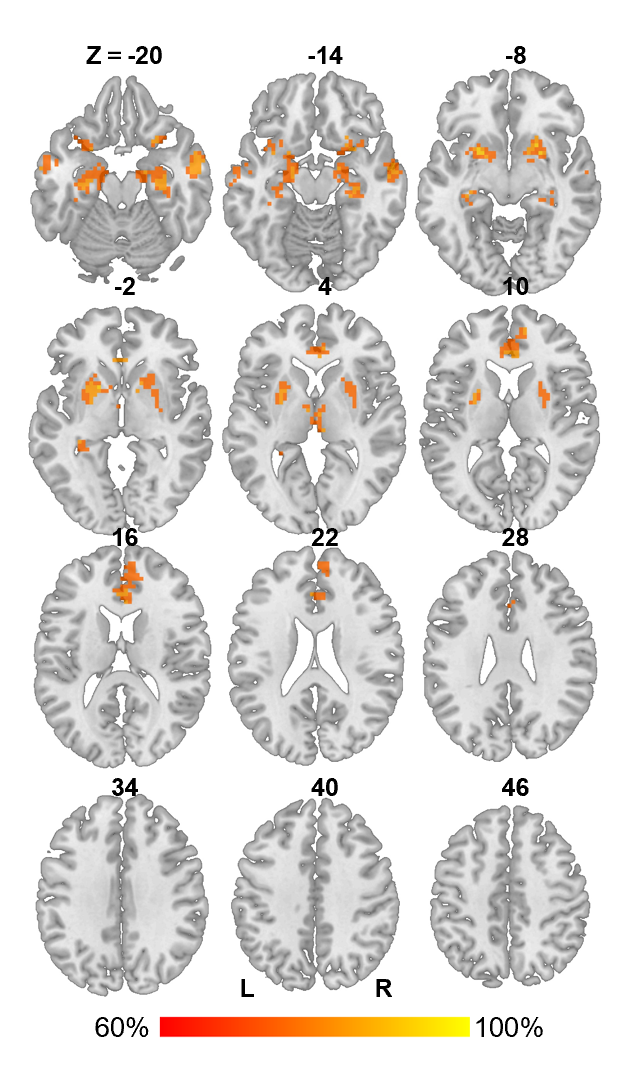


**Figure S4.** The COVID-19 survivors’ dysfunctional networks based on 7-mm radius sphere. The dysfunctional network is presented as network probability maps thresholded at 60%, showing brain regions functionally connected to more than 60% of the contrast seeds. Abbreviations: L, left; R, right; SALD, the Southwest University Adult Lifespan Dataset.


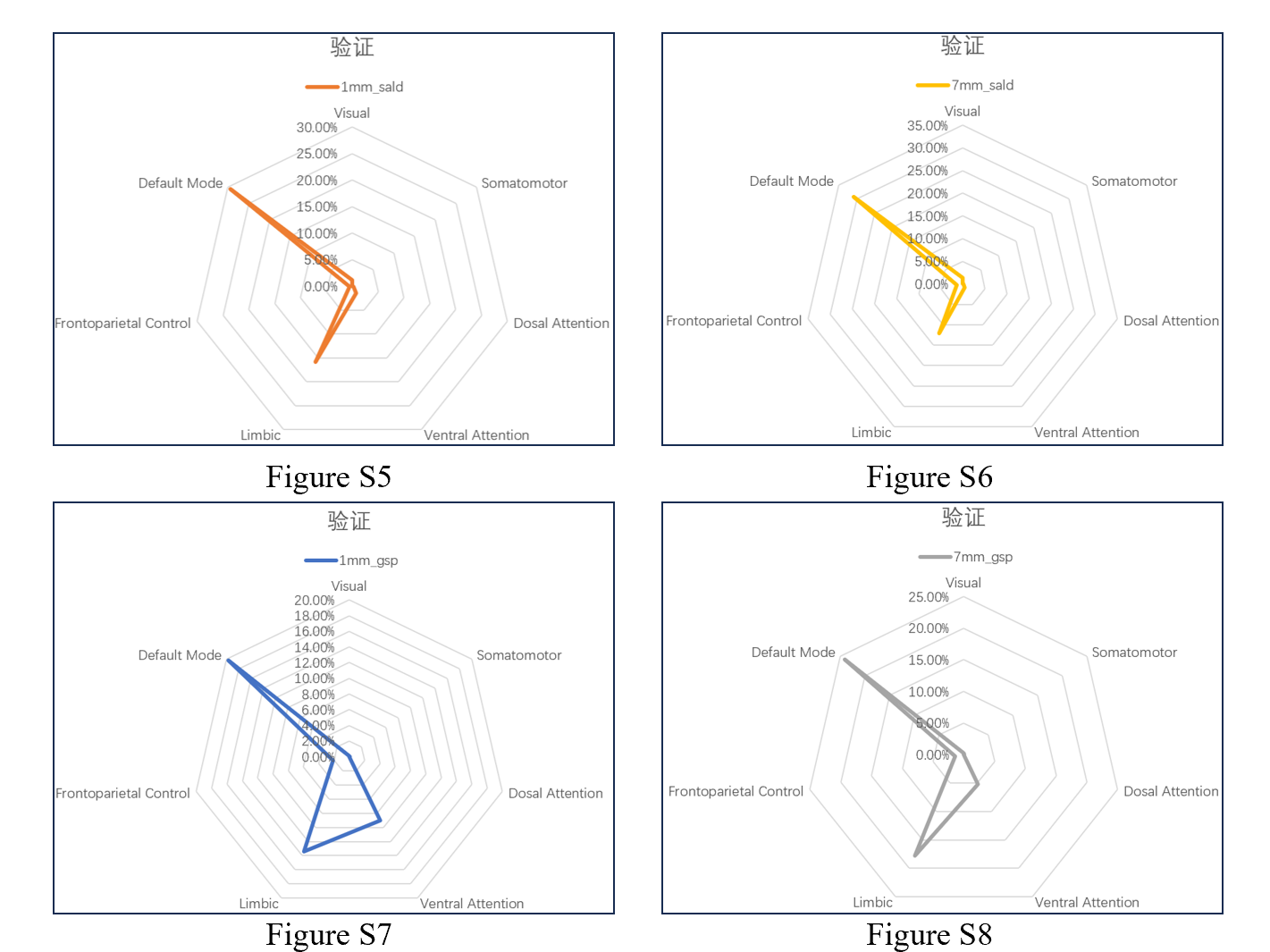


**Figure S5-8.** COVID-19 survivors’ dysfunctional networks in relation to canonical brain networks, respectively with sphere radius of 1 mm in SALD data, sphere radius of 7 mm in SALD data, sphere radius of 1 mm in GSP data, sphere radius of 7 mm in GSP data. Polar plots illustrate the proportion of overlapping voxels between survivors’ dysfunctional network and a canonical network to all voxels within the corresponding canonical network.

Ajcevic, M., K. Iscra, G. Furlanis, M. Michelutti, A. Miladinovic, A. Buoite Stella, M. Ukmar, M. A. Cova, A. Accardo and P. Manganotti (2023). "Cerebral hypoperfusion in post-COVID-19 cognitively impaired subjects revealed by arterial spin labeling MRI." Sci Rep **13**(1): 5808.

Besteher, B., M. Machnik, M. Troll, A. Toepffer, A. Zerekidze, T. Rocktaschel, C. Heller, Z. Kikinis, S. Brodoehl, K. Finke, P. A. Reuken, N. Opel, A. Stallmach, C. Gaser and M. Walter (2022). "Larger gray matter volumes in neuropsychiatric long-COVID syndrome." Psychiatry Res **317**: 114836.

Bispo, D. D. C., P. R. P. Brandao, D. A. Pereira, F. B. Maluf, B. A. Dias, H. R. Paranhos, F. von Glehn, A. C. P. de Oliveira, N. A. T. Regattieri, L. S. Silva, C. L. Yasuda, A. Soares and M. Descoteaux (2022). "Brain microstructural changes and fatigue after COVID-19." Front Neurol **13**: 1029302.

Chang, L., M. C. Ryan, H. Liang, X. Zhang, E. Cunningham, J. Wang, E. Wilson, E. H. Herskovits, S. Kottilil and T. M. Ernst (2023). "Changes in Brain Activation Pattern During Working Memory Tasks in People With Post-COVID Condition and Persistent Neuropsychiatric Symptoms." Neurology.

Díez-Cirarda, M., M. Yus, N. Gómez-Ruiz, C. Polidura, L. Gil-Martínez, C. Delgado-Alonso, M. Jorquera, U. Gómez-Pinedo, J. Matias-Guiu, J. Arrazola and J. A. Matias-Guiu (2023). "Multimodal neuroimaging in post-COVID syndrome and correlation with cognition." Brain **146**(5): 2142-2152.

Donegani, M. I., A. Miceli, M. Pardini, M. Bauckneht, S. Chiola, M. Pennone, C. Marini, F. Massa, S. Raffa, G. Ferrarazzo, D. Arnaldi, G. Sambuceti, F. Nobili and S. Morbelli (2021). "Brain Metabolic Correlates of Persistent Olfactory Dysfunction after SARS-Cov2 Infection." Biomedicines **9**(3).

Du, Y., W. Zhao, S. Huang, Y. Huang, Y. Chen, H. Zhang, H. Guo and J. Liu (2023). "Two-year follow-up of brain structural changes in patients who recovered from COVID-19: A prospective study." Psychiatry Res **319**: 114969.

Du, Y. Y., W. Zhao, X. L. Zhou, M. Zeng, D. H. Yang, X. Z. Xie, S. H. Huang, Y. J. Jiang, W. H. Yang, H. Guo, H. Sun, J. Y. Liu, P. Liu, Z. G. Zhou, H. Luo and J. Liu (2022). "Survivors of COVID-19 exhibit altered amplitudes of low frequency fluctuation in the brain: a resting-state functional magnetic resonance imaging study at 1-year follow-up." Neural Regen Res **17**(7): 1576-1581.

Goehringer, F., A. Bruyere, M. Doyen, S. Bevilacqua, A. Charmillon, S. Heyer and A. Verger (2023). "Brain (18)F-FDG PET imaging in outpatients with post-COVID-19 conditions: findings and associations with clinical characteristics." Eur J Nucl Med Mol Imaging **50**(4): 1084-1089.

Guedj, E., J. Y. Campion, P. Dudouet, E. Kaphan, F. Bregeon, H. Tissot-Dupont, S. Guis, F. Barthelemy, P. Habert, M. Ceccaldi, M. Million, D. Raoult, S. Cammilleri and C. Eldin (2021). "(18)F-FDG brain PET hypometabolism in patients with long COVID." Eur J Nucl Med Mol Imaging **48**(9): 2823-2833.

Hafiz, R., T. K. Gandhi, S. Mishra, A. Prasad, V. Mahajan, X. Di, B. H. Natelson and B. B. Biswal (2022). "Higher limbic and basal ganglia volumes in surviving COVID-negative patients and the relations to fatigue." Neuroimage Rep **2**(2): 100095.

Kamasak, B., T. Ulcay, M. Nisari, O. Gorgulu, V. Akca, M. Alpaslan, A. Yetis, L. Hizmali, M. K. Karahocagil and K. Aycan (2023). "Effects of COVID-19 on brain and cerebellum: a voxel based morphometrical analysis." Bratisl Lek Listy **124**(6): 442-448.

Kas, A., M. Soret, N. Pyatigoskaya, M. O. Habert, A. Hesters, L. Le Guennec, O. Paccoud, S. Bombois, C. Delorme, g. on the behalf of CoCo-Neurosciences study and C. S. P. s. group (2021). "The cerebral network of COVID-19-related encephalopathy: a longitudinal voxel-based 18F-FDG-PET study." Eur J Nucl Med Mol Imaging **48**(8): 2543-2557.

Kim, W. S. H., X. Ji, E. Roudaia, J. J. Chen, A. Gilboa, A. Sekuler, F. Gao, Z. Lin, A. Jegatheesan, M. Masellis, M. Goubran, J. S. Rabin, B. Lam, I. Cheng, R. Fowler, C. Heyn, S. E. Black, S. J. Graham and B. J. MacIntosh (2022). "MRI Assessment of Cerebral Blood Flow in Nonhospitalized Adults Who Self-Isolated Due to COVID-19." J Magn Reson Imaging.

Li, R., G. Liu, X. Zhang, M. Zhang, J. Lu and H. Li (2023). "Altered intrinsic brain activity and functional connectivity in COVID-19 hospitalized patients at 6-month follow-up." BMC Infect Dis **23**(1): 521.

Morand, A., J. Y. Campion, A. Lepine, E. Bosdure, L. Luciani, S. Cammilleri, B. Chabrol and E. Guedj (2022). "Similar patterns of [(18)F]-FDG brain PET hypometabolism in paediatric and adult patients with long COVID: a paediatric case series." Eur J Nucl Med Mol Imaging **49**(3): 913-920.

Qin, Y., J. Wu, T. Chen, J. Li, G. Zhang, D. Wu, Y. Zhou, N. Zheng, A. Cai, Q. Ning, A. Manyande, F. Xu, J. Wang and W. Zhu (2021). "Long-term microstructure and cerebral blood flow changes in patients recovered from COVID-19 without neurological manifestations." J Clin Invest **131**(8).

Tu, Y., Y. Zhang, Y. Li, Q. Zhao, Y. Bi, X. Lu, Y. Kong, L. Wang, Z. Lu and L. Hu (2021). "Post-traumatic stress symptoms in COVID-19 survivors: a self-report and brain imaging follow-up study." Mol Psychiatry **26**(12): 7475-7480.

Wingrove, J., J. Makaronidis, F. Prados, B. Kanber, M. C. Yiannakas, C. Magee, G. Castellazzi, L. Grandjean, X. Golay, C. Tur, O. Ciccarelli, E. D'Angelo, C. A. M. Gandini Wheeler-Kingshott and R. L. Batterham (2023). "Aberrant olfactory network functional connectivity in people with olfactory dysfunction following COVID-19 infection: an exploratory, observational study." EClinicalMedicine **58**: 101883.
